# Rolling Into Older Adulthood: Illustrating Cumulative (Dis)Advantage With a Dice-Based Role Playing Game

**DOI:** 10.1093/geroni/igaf122.1587

**Published:** 2025-12-31

**Authors:** L B Bouchard

**Affiliations:** Western Oregon University, Monmouth, Oregon, United States

## Abstract

Undergraduate curriculum can benefit from active, creative classroom participation aligned with the Academy for Gerontology in Higher Education (AGHE) competencies. In order to achieve student comprehension of AGHE standard I. 4. 2 (i.e., assess the impact of inequality on individual and group life opportunities throughout the lifespan/course impacting late-life outcomes), a role playing game (RPG) was developed. This activity was designed for an upper level undergraduate course called Intersectionality: Inequalities and Vulnerabilities in Older Adulthood. RPGs are being incorporated into higher education curriculum to encourage engaged student learning, foster empathy, and promote an understanding of deeper level concepts. RPGs are also useful to solidify challenging concepts while encouraging deeper social interaction, and this pedagogical modality lends itself to complex life narratives. Students begin by rolling for a case study, and each group is randomly assigned a personal history of a five year old child. Utilizing dice from table top role playing games, such as Dungeons and Dragons, students progress through the journey of life in adulthood with a combination of chance, choice, and circumstances outside their control. The instructor takes an active role to guide the scenarios, elicit reactions, and determine the outcome of student decision making. This presentation will focus on the basics of RPGs in higher education, explain instructions and parameters for this competency based activity, and explain next steps for instructors implementing this activity or other RPGs into their gerontology courses.

